# Nutritional alterations, adverse consequences, and comprehensive assessment in spinal cord injury: a review

**DOI:** 10.3389/fnut.2025.1576976

**Published:** 2025-05-09

**Authors:** Zehui Li, Xiaoxin Wang, Yan Yu, Yingli Jing, Huayong Du, Wubo Liu, Chunjia Zhang, Zuliyaer Talifu, Xin Xu, Yunzhu Pan, Jianjun Li

**Affiliations:** ^1^School of Rehabilitation, Capital Medical University, Beijing, China; ^2^Department of Spinal and Neural Functional Reconstruction, China Rehabilitation Research Center, Beijing, China; ^3^China Rehabilitation Science Institute, Beijing, China; ^4^Department of Orthopaedics, Qilu Hospital, Cheeloo College of Medicine, Shandong University, Shandong, China; ^5^Department of Rehabilitation Medicine, Peking University Third Hospital, Beijing, China; ^6^School of Population Medicine and Public Health, Chinese Academy of Medical Sciences/Peking Union Medical College, Beijing, China; ^7^Rehabilitation Department, Beijing Hospital, National Center of Gerontology, Institute of Geriatric Medicine, Chinese Academy of Medical Sciences, Beijing, China

**Keywords:** spinal cord injury, nutritional status, nutritional assessment, body composition, energy imbalance, complications, metabolic alterations

## Abstract

Spinal cord injury (SCI) leads to complex nutritional alterations, including energy imbalance, skewed macronutrient and micronutrient intake, and disrupted nutrient absorption and metabolism. These changes contribute to increased risks of obesity, cardiovascular disease, metabolic syndrome, and other comorbidities, profoundly affecting long-term recovery and quality of life. Despite the growing recognition of these challenges, nutritional assessment methods for SCI patients remain fragmented and insufficient. This review first outlines the major nutritional consequences and clinical implications of SCI, then focuses on current methods for assessing nutritional status in this population. Three major domains are discussed: body composition analysis, nutrient intake and absorption assessment, and energy metabolism monitoring. Traditional tools such as anthropometry, food diaries, and indirect calorimetry are discussed alongside advanced technologies including magnetic resonance imaging (MRI), dual-energy X-ray absorptiometry (DXA), and metabolomics. By highlighting both current limitations and emerging solutions, this review underscores the importance of personalized, technology-assisted nutritional assessment strategies to guide clinical decision-making and optimize outcomes for individuals with SCI.

## 1 Introduction

Spinal cord injury (SCI) refers to spinal cord dysfunction caused by trauma or disease, leading to partial or complete loss of motor, sensory, and autonomic functions. This injury is often irreversible and significantly reduces the quality of life for affected individuals (1–3). Recently published international guidelines and expert consensus statements consistently recognize that approximately 50% of SCI patients experience complex nutritional changes that profoundly affect metabolic function and significantly increase the risk of obesity and related complications (4–6). Research has shown that the imbalance between energy intake and expenditure due to SCI differs across injury phases and types. For example, the acute phase presents high metabolic demands, which may shift to energy excess in the chronic phase, increasing the risk of body fat accumulation and metabolic disorders (2, 7–9).

Additionally, imbalances in macronutrient (such as carbohydrates, proteins, and fats) and micronutrient(including vitamins and minerals) intake complicate weight management and metabolic regulation. Specifically, diets high in carbohydrates and fats, low in protein, and deficient in micronutrients require urgent attention (2, 5, 7, 10). SCI is also associated with gut dysbiosis, a condition increasingly recognized in affected individuals (11). Although the exact mechanisms remain under investigation, current evidence suggests that impaired autonomic control and reduced gastrointestinal motility following SCI may contribute to alterations in gut microbiota composition (10, 12). These changes can disrupt nutrient absorption and metabolism, thereby further impacting both digestive and neurological function (11, 13, 14). Changes in body composition—such as increased body fat and decreased lean body mass(LBM)—exacerbate neurogenic obesity and bone loss (15, 16), while fatigue affects rehabilitation participation and outcomes (17, 18). Moreover, multiple comorbidities like malnutrition, infections, and stress injuries are linked to poor nutritional status, adding to the overall health burden (8, 19, 20).

Given the variability in injury characteristics, dietary habits, and compliance among SCI patients, managing nutritional needs becomes complex (8, 21). Therefore, accurate monitoring and assessment of nutritional status, including body composition, nutrient intake, absorption, and energy metabolism, is essential for optimal management of comorbidities and improving patients’ quality of life (8, 22–24). By combining advanced assessment tools with individualized interventions, SCI patients’ nutritional issues can be more effectively managed, promoting recovery and long-term health.

This narrative review aims to: (1) examine the impact of SCI on nutritional changes, summarizing imbalances in energy and nutrient intake at different stages and across injury types. It will focus on how these imbalances affect weight management and metabolic regulation, emphasizing the importance of dietary interventions. (2) Address the challenges posed by changes in body composition, neurogenic obesity, bone loss, and other comorbidities. (3) Evaluate methods for assessing nutritional status in SCI patients. Ultimately, this review seeks to provide a scientific foundation for managing comorbidities and improving the quality of life for SCI patients, offering strategies for better nutritional management, rehabilitation, and health maintenance.

## 2 Nutritional alterations after spinal cord injury

Patients with SCI often experience various nutritional changes that can negatively impact their body composition and increase the risk of metabolic disorders and other complications. The following discussion focuses on changes in energy intake, energy expenditure, macronutrient and micronutrient levels to provide a comprehensive overview of the nutritional alterations associated with SCI.

### 2.1 Energy imbalance after SCI

Patients with SCI typically face an imbalance between energy intake and expenditure, which varies in the acute and chronic phases of injury and is influenced by several factors. Understanding the dynamics of this imbalance is crucial for managing rehabilitation effectively. This section will explore energy intake and expenditure separately, as well as the long-term health implications of these imbalances.

#### 2.1.1 Energy intake

Energy intake in SCI patients is influenced by factors such as age, gender, injury type, severity, and mobility. Studies show that the daily caloric intake for SCI patients is generally similar to or slightly lower than that of the general population (1,800 to 2,600 kcal/day) (7, 8). However, energy intake tends to be lower in older patients, females, and those with longer-term injuries (25, 26). During the acute phase, SCI patients typically have a higher energy intake compared to the recovery phase and to patients with other neurological injuries, such as traumatic brain injury (27). In the first 4 weeks after injury, energy intake may increase by more than 400 kcal/day, likely due to increased metabolic stress, inflammation, and protein breakdown. Moreover, the thermic effect of respiration for patients off the ventilator also raises energy requirements. The degree of injury further influences energy intake, with paraplegic patients generally consuming more calories than tetraplegic patients, although differences in research methodology can impact these findings (22, 25, 26).

#### 2.1.2 Energy expenditure

Total daily energy expenditure (TDEE) in SCI patients is primarily determined by basal metabolic rate (BMR), thermic effect of food (TEF), and thermic effect of physical activity (TEPA), with BMR being the most significant component. TEPA, which varies depending on LBM and activity levels, is the most volatile aspect of energy expenditure (28). SCI patients with quadriplegia or complete injuries typically have lower TEPA and BMR compared to those with paraplegia or incomplete injuries (29–32). During the acute phase, metabolic stress and tissue repair demands cause a substantial increase in TDEE (33, 34). However, in the chronic phase, energy expenditure decreases significantly—up to 54% in quadriplegic patients and 20% in paraplegic patients (5). This suggests that dietary intake must be adjusted throughout the injury phases to prevent energy imbalances. Despite exercise interventions increasing TDEE and BMR, the high percentage of body fat in these patients remains, indicating that exercise alone is insufficient to improve body composition and must be paired with dietary adjustments (34).

#### 2.1.3 Energy imbalance

The imbalance between energy intake and expenditure can have significant long-term health consequences. During the acute phase, SCI patients may experience negative nitrogen balance due to the increased metabolic demands of injury (8, 9). However, in the chronic phase, daily energy intake (1,516–2,150 kcal/day) typically exceeds energy expenditure (1,414–1,569 kcal/day) (2, 7), leading to energy surplus. Over time, this surplus contributes to weight gain, fat accumulation, and an elevated risk of secondary health conditions, including cardiovascular disease, insulin resistance, and metabolic syndrome.

Measurement methods for energy intake and expenditure in SCI patients have limitations, particularly due to reliance on recall questionnaires and self-reports, which can introduce inaccuracies. The heterogeneity of patient populations—such as differences in injury severity and duration—further complicates comparisons across studies. Future research should employ more standardized quantitative tools and multicenter designs to improve the accuracy and comparability of results.

In conclusion, energy intake and expenditure in SCI patients vary significantly across injury stages and types. While acute-phase energy demands are high, these eventually shift to a risk of energy surplus in the chronic phase, promoting fat accumulation and metabolic disturbances. Existing studies on energy balance are hindered by measurement challenges and patient variability, highlighting the need for standardized methodologies. Effective management of energy balance is crucial for preventing complications and optimizing rehabilitation, with the quality of macronutrient intake—beyond just total calorie consumption—playing a key role in maintaining health. This aspect will be explored further in the following sections.

### 2.2 Imbalanced nutrient intake after SCI

**Figure 1:**
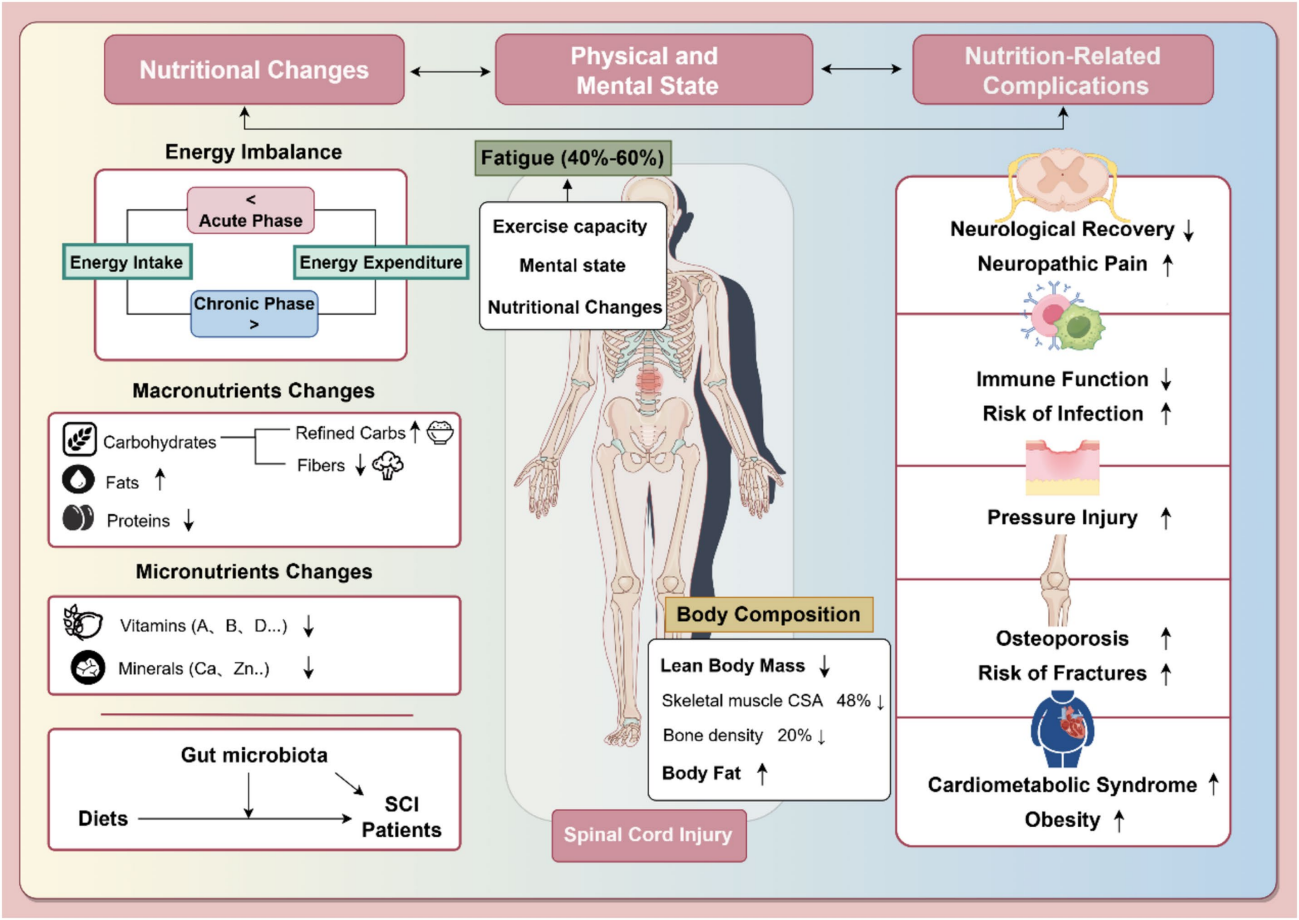
Nutritional changes after spinal cord injury and their impact on health status and nutrition-related complications. The left section details nutritional imbalances in SCI, such as altered energy balance, macronutrient changes, and micronutrient deficits, alongside the gut microbiota’s role. The middle section highlights body composition changes (lean body mass, body fat) and associated fatigue, affecting 40–60% of SCI patients. The right section outlines nutrition-related complications, including neurological impairments, immune dysfunction, pressure injuries, osteoporosis, and cardiometabolic syndrome. Arrows indicate trends: “↑” for increases and “↓” for decreases. Part of the figure was created using FigDraw.com.

Patients with SCI often exhibit significant imbalances in their nutrient intake, characterized by both inadequate and excessive consumption of macronutrients and micronutrients (Figure 1). These dietary imbalances not only increase the risk of metabolic syndrome and cardiovascular disease but also adversely affect weight management, metabolic regulation, bone health, and overall quality of life (8).

#### 2.2.1 Macronutrient intake imbalances

##### 2.2.1.1 Carbohydrates

Carbohydrate intake in SCI patients is typically high, averaging 969 kcal/day (95% CI: 851–1,087) in chronic SCI cases (7), far exceeding the USDA 2020–2025 Dietary Guidelines, which recommend carbohydrate intake contributing 45–65% of total daily energy (approximately 225–325 g/day based on a 2,000 kcal diet). This high carbohydrate intake is inconsistent with the decreased energy expenditure observed in SCI patients, exacerbating challenges in weight management. Patients tend to consume an excessive amount of simple carbohydrates while under-consuming complex carbohydrates and dietary fiber. As a result, their dietary fiber intake averages only 17 g/day, significantly lower than the recommended 25–34 g/day for adults. This fiber deficiency contributes to metabolic imbalances and worsens symptoms of neurogenic bowel dysfunction, such as constipation and prolonged intestinal transit time. A gradual increase in dietary fiber to 30 g/day is recommended for chronic SCI patients, but this should be accompanied by adequate fluid intake to avoid worsening bowel symptoms (10, 35).

##### 2.2.1.2 Protein

Daily protein intake in chronic SCI patients averages 319 kcal (95% CI: 294–345) (7), exceeding the USDA recommendation that protein should account for 10%–35% of total daily calories (approximately 50–175 g/day for a 2,000 kcal diet). While protein intake generally meets or exceeds recommendations, it may decline with weight gain during the chronic phase, particularly in obese patients (36). One study found that protein and fat intake were higher in quadriplegic patients compared to paraplegic patients (33). However, this higher intake does not prevent the risk of neurogenic obesity, which is more common in patients with lower activity levels. Interestingly, despite higher protein intake, some obese SCI patients still suffer from insufficient protein consumption relative to their caloric intake, suggesting a complex relationship between obesity, protein intake, and dietary structure. Excessive protein intake may also negatively affect bone mineral density (BMD) in the lumbar spine, though no significant effects have been noted for the femur or hip. Therefore, a balanced approach considering both body composition and bone health is essential when managing protein intake.

##### 2.2.1.3 Fat

Fat intake in chronic SCI patients averages 663 kcal/day (95% CI: 590–736), representing 34–40% of total energy intake (7), which exceeds the USDA recommended range of 20–35%. More concerning is the source of fat intake, particularly the high consumption of saturated fatty acids (SFAs). The USDA recommends replacing SFAs with monounsaturated (MUFA) and polyunsaturated fatty acids (PUFA) to promote cardiovascular health. However, SCI patients, particularly those with paraplegia and quadriplegia, tend to exceed recommended levels of SFA intake (8). High SFA consumption is linked to increased risks of cardiovascular disease and all-cause mortality (37, 38). The Paralyzed Veterans of America recommends limiting SFA intake to 5–6% of total energy in SCI patients to reduce the risk of cardiovascular disease. Additionally, MUFA intake tends to be higher in patients with incomplete injuries compared to those with complete injuries, and PUFA metabolism is disrupted in the spinal cord following SCI, leading to omega-6 fatty acid imbalance and omega-3 (specifically docosahexaenoic acid (DHA)) deficiency. This imbalance contributes to neurological dysfunction, including pain and motor issues, and may be alleviated by a diet rich in omega-3 PUFA (39–41).

#### 2.2.2 Micronutrient intake imbalances

Micronutrient deficiencies are common in chronic SCI patients, with many suffering from insufficient levels of vitamins A, B5, B7, B9, D, E, and minerals such as potassium and calcium. Conversely, excessive intake of vitamins B1, B2, B3, B12, C, K, sodium, phosphorus, copper, and zinc has been noted (7). Vitamin D deficiency, in particular, is widespread, especially in the winter months, and even elite SCI athletes face this issue due to reduced outdoor activity. Prolonged vitamin D deficiency often results in sublesional bone loss, increasing the risk of osteoporosis and fractures (42, 43). Additionally, the majority of SCI patients have inadequate calcium intake, which, combined with reduced dairy consumption, exacerbates bone health issues (44–46). To mitigate these risks, adequate intake of vitamin D and calcium should be a nutritional priority in SCI management.

Beyond vitamin D, the homeostatic balance of other vitamins plays an equally crucial role in the rehabilitation of individuals with SCI, influencing a range of physiological processes such as immune regulation, oxidative stress control, and neural repair. For example, vitamin A has been shown to promote neural regeneration and exerts neuroprotective effects in SCI animal models (47). B-complex vitamins have been found to inhibit apoptosis, enhance remyelination, and improve lipid metabolism (48, 49). Vitamin C contributes to regulate inflammation and oxidative stress (50), while vitamin E exerts neuroprotective effects by modulating immune responses (51). Although emerging clinical evidence supports the potential benefits of systemic vitamin supplementation (52), the optimal dosage and administration regimens remain to be established through large-scale randomized controlled trials.

Mineral imbalances may likewise hinder functional recovery after SCI. Trace elements such as zinc and selenium are vital for the regulation of oxidative stress and neural remodeling via modulation of peroxidase activity (53, 54). Major electrolytes—sodium, potassium, magnesium, and calcium—function synergistically to maintain nerve conduction, muscle contraction, and bone metabolism. These multisystem regulatory functions are particularly relevant to SCI, which affects neural, motor, and autonomic systems (7, 21, 44). It is important to emphasize that mineral supplementation must follow individualized dosing principles, as both deficiencies and excesses can disrupt internal homeostasis and increase the risk of secondary complications.

Given the complexity of micronutrient imbalances and their wide-ranging impacts post-injury, personalized monitoring and targeted dietary planning should be integrated into routine SCI management. Such strategies are essential for supporting metabolic, musculoskeletal, and neurological recovery throughout the rehabilitation process.

In summary, SCI patients typically have diets high in carbohydrates and fats but low in protein, fiber, and essential micronutrients. These imbalances contribute to difficulties in weight management, metabolic regulation, cardiovascular health, bone density, and neurological function. Given these challenges, understanding the nutritional implications and complications of SCI is crucial for effective management and rehabilitation.

## 3 Nutritional implications and complications in SCI patients

### 3.1 Physical and mental status after SCI

Nutritional changes following SCI lead to significant alterations in body composition, particularly an increase in body fat and a decrease in lean body mass (LBM). Studies show that neurogenic obesity affects between 22 and 97% of adult SCI patients, contributing to dysfunctional energy metabolism, reduced physical adaptation, sympathetic nervous system dysfunction, hormonal abnormalities, altered satiety, and loss of LBM (5, 55). Bone loss is another rapid consequence of SCI, with bone mineral density at the knee and hip decreasing by 2 to 4% per month (56, 57), totaling approximately 20% within the first year of injury (15, 58). Skeletal muscle mass below the injury site also diminishes significantly, with cross-sectional area decreasing by as much as 48% within the first 6 weeks (59). Skeletal muscle atrophy contributes to 30 to 60% of total LBM loss in SCI patients (16). Given these reductions in LBM, many SCI patients are classified as overweight or obese based on body fat percentage (55).

In individuals with SCI, the coexistence of obesity and malnutrition—two seemingly contradictory conditions—has emerged as a significant clinical concern. This dual risk, often referred to as “obesity–malnutrition,” arises from a complex interplay of physiological, psychological, and social factors. On the one hand, imbalanced dietary intake can lead to excess caloric consumption, particularly when increased protein intake is not offset by a reduction in other macronutrients, thereby increasing the risk of neurogenic obesity (60). On the other hand, neurogenic obesity itself can further disrupt metabolic processes, exacerbating nutritional deficiencies (35).

Beyond metabolic dysregulation and reduced physical activity inherent to SCI, psychosocial and functional limitations also play a critical role in the development of this dual nutritional burden. Psychological disorders are highly prevalent among individuals with SCI (61), and may reduce engagement in outdoor activities and rehabilitation programs (62), thus lowering overall energy expenditure. Functional impairments—such as wheelchair dependence and compromised hand function—can limit the ability to shop for, prepare, and store nutritious foods, fostering reliance on low-quality, energy-dense convenience foods (63). Additionally, changes in food-related social dynamics, feelings of loneliness and shame, and a diminished sense of autonomy during assisted feeding may not only affect nutritional intake but also negatively alter the overall eating experience (64).

Compounding these issues, approximately 40 to 60% of individuals with SCI report moderate to severe fatigue, which further interferes with daily functional activities and hinders participation in rehabilitation (17). Fatigue in this population has been linked to reduced motor performance, psychological distress, and nutritional imbalances (18, 65), reinforcing a vicious cycle in which physical and mental decline contribute to poor dietary outcomes, which in turn perpetuate both malnutrition and obesity.

### 3.2 SCI comorbidities from a nutritional perspective

Autonomic dysfunction following SCI disrupts the sympathetic, parasympathetic, and enteric nervous systems, leading to impaired gastrointestinal motility, digestion, and nutrient absorption (12). These effects vary based on injury severity and may persist for years, significantly compromising nutritional status (12, 66). The resulting nutritional alterations are closely associated with a range of comorbidities that directly affect recovery and quality of life.

Malnutrition is common, with studies showing that 62% of SCI patients are at risk of malnutrition 3 months post-injury, with this risk remaining as high as 40% at discharge (67). Malnutrition is strongly correlated with poor neurological recovery and increased mortality, particularly among older patients (3). It weakens immune function, increasing susceptibility to infections and prolonging recovery time. SCI patients, especially those at risk for malnutrition, also face a high incidence of urinary tract infections, which affect up to 50% of these patients (68).

Stress injuries, such as decubitus ulcers, are common in SCI patients and are often exacerbated by poor nutritional status. Inadequate protein and energy intake contribute to impaired skin healing, while static postures due to reduced mobility further increase the risk of pressure ulcers. Studies show that appropriate nutritional interventions can improve wound healing and reduce the risk of pressure injuries (69). Additionally, SCI patients often experience reduced physical activity, further increasing the risk of obesity and metabolic syndrome. The presence of metabolic syndrome, along with chronic systemic inflammation caused by excessive body fat, may heighten the risk of neuropathic pain (70). Furthermore, the combination of increased body fat and decreased muscle mass may predispose SCI patients to chronic diseases such as type 2 diabetes and cardiovascular disease (6, 71).

An emerging issue in SCI patients is the gut microbiota dysbiosis. SCI can impair autonomic control of the gastrointestinal system, leading to slower transit time and increased intestinal permeability—both of which are known contributors to microbial imbalance (10, 12). Additionally, factors such as reduced mobility, altered diet, and frequent antibiotic use further promote dysbiosis in this population (65). Such dysbiosis of the gut microbiota has been linked to systemic inflammation and metabolic disturbances, potentially contributing to the aggravation of other SCI-related complications (11, 13, 14). Nutritional changes after SCI are closely linked to alterations in gut microbiota composition and metabolism, which affect the intake and metabolism of nutrients. Dysbiosis can also impact the host through the gut–brain axis, exacerbating neurogenic bowel dysfunction and other related problems. These changes in gut function, including altered digestion, absorption, and appetite, indirectly worsen nutritional problems after SCI (72–74). The interaction between dietary changes and gut dysbiosis is complex and under-researched, warranting further investigation into the mechanisms at play (13, 66). This relationship could help develop more effective strategies for addressing the nutritional challenges faced by SCI patients.

In summary, SCI patients face a range of nutritional complications, including imbalances in body composition, malnutrition, and comorbidities such as infections, stress injuries, and metabolic syndrome (Figure 1). These complications not only hinder recovery but also pose long-term health risks. Addressing nutritional imbalances through tailored interventions is essential for improving the overall health and rehabilitation outcomes of SCI patients. Furthermore, the connection between gut microbiota dysbiosis and nutritional changes highlights the need for a holistic approach to managing SCI patients’ nutritional needs, incorporating both dietary adjustments and gut health interventions.

## 4 Nutritional systems assessment methods for spinal cord injury

Nutritional assessment in patients with SCI is a comprehensive and systematic process designed to identify nutritional issues and their underlying causes and severity. This assessment involves the collection and analysis of relevant data to guide individualized interventions. Continuous monitoring ensures that these interventions remain effective and aligned with the patient’s evolving needs. A thorough nutritional assessment typically includes evaluations of body composition, nutrient intake and absorption, and energy metabolism (Table 1). Given the unique physiological changes and metabolic disturbances resulting from SCI, conventional assessment methods may not fully capture the specific needs of these patients. Therefore, these methods must be complemented by specialized clinical tools and technologies to provide a more accurate and detailed picture of a patient’s nutritional status. This integrated approach enables the development of more effective health management strategies tailored to the individual.

**Table 1:** Comparison of systematic nutritional assessment methods for patients with spinal cord injury.

Evaluation dimension	Method/tool	Key indicators	Advantages	Limitations	References
Body composition assessment	Basic Anthropometric Tools	Height, weight, skinfold thickness, body and limb circumferences (waist, arm circumference, etc.) and derived indices (BMI, WHtR, WHR)	Non-invasive; low cost; easy to operate; rapid screening; widely used in clinical practice	Primarily for initial assessment, not suitable for detailed evaluation; measurement methods need to be adjusted for SCI patients; controversial screening and predictive effectiveness for some indicators	(23, 75, 76, 77, 79, 82)
	Ultrasound	Muscle length, thickness, CSA, and other muscle morphology data; blood flow parameters, vascular morphology data	Non-invasive, easy to operate, real-time dynamic observation, high portability, multi-parameter evaluation	High operator dependence; limited depth and field of view; low image resolution; cannot provide comprehensive assessment	(89)
	MRI	Fat distribution, more accurate measurements of waist, arm circumference, etc.; especially the length, CSA of individual muscles	Non-invasive; high soft tissue resolution; quantifiable	Limited by space, cannot be performed at the bedside; high cost	(88)
	Dual-Energy X-ray Absorptiometry (DXA)	Data on fat, muscle, and bone density	Gold standard, high repeatability	Radiation exposure limits frequent use; expensive equipment; high cost	(79)
	Bioelectrical Impedance Analysis	Fat content, body fat percentage, LBM, water content	Non-invasive, convenient, suitable for fat mass assessment (especially in women and individuals with impaired mobility)	Prediction errors in body fat percentage for SCI patients need calibration; general equations for male cervical SCI patients’ FFM assessment may be biased, specific equations needed	(90, 91)
Nutritional intake and absorption assessment	24-h Dietary Recall and Food Frequency Questionnaire (FFQ)	Dietary intake and types, dietary habits	Simple and convenient; comprehensive assessment; low cost; short-term intake evaluation, reveals dietary patterns and nutritional needs	High subjectivity, dependent on patient memory; difficult to describe special diets; inaccurate quantification; FFQ categories limited; poor SCI specificity	(92, 93, 94)
	SCI-specific Nutritional Screening Tool (SNST)	Weight changes, dietary intake, gastrointestinal function, SCI condition	Simple and convenient; highly applicable; optimizes muscle wasting and resting energy expenditure assessment; more accurate in identifying high-risk malnutrition patients; higher sensitivity and negative predictive value than traditional screening tools	High subjectivity; lack of standardization; low adoption rate	(96, 97)
	Simplified Nutritional Appetite Questionnaire (SNAQ)	Appetite status, meal frequency, satiety	Simple and convenient; rapid screening; assesses appetite characteristics, complements nutritional status assessment	High subjectivity; lacks specificity	(99, 100)
	Stable Isotope Labeling Techniques (e.g., ^13^C-Leucine)	Nutrient absorption efficiency (e.g., fat absorption), tissue utilization efficiency	Accurate quantification of nutrient absorption efficiency, reveals metabolic disruption mechanisms	Complex operation, high cost	(101, 102)
	Intestinal Permeability Testing (e.g., Lactulose/Mannitol permeability test, tight junction biomarkers)	Monitoring of substance intake and absorption (e.g., lactulose, mannitol)	Simple; non-invasive; reflects changes in intestinal permeability, assesses nutrient absorption	High technical requirements; complex result interpretation; significant individual variability	(103)
	Metabolomics Techniques (LC–MS/MS, ^1^H NMR)	Metabolic profile changes of SCI-specific metabolites, indirectly assesses breakdown and absorption efficiency	Analyzes nutritional metabolism pathways, identifies SCI-specific metabolites (e.g., acetylphosphate)	Exploratory tool, clinical translation requires validation	(105, 106, 107)
Energy metabolism assessment	Indirect Calorimetry	Basal metabolic rate (BMR), resting energy expenditure (REE), respiratory quotient (RQ), oxygen consumption (VO2)	Gold standard, accurately measures REE; analyzes energy expenditure composition	Expensive equipment, low availability	(109, 110)
	Doubly Labeled Water Method	Total energy expenditure (TEE), carbon dioxide production	High precision; non-invasive; long-term monitoring; suitable for a wide population	Complex sample processing; high cost; low availability; cannot differentiate energy expenditure composition	(111, 112)
	Wearable Sensors (Accelerometers)	TEE, energy expenditure during physical activity	Dynamic monitoring of energy expenditure in SCI patients, especially wheelchair users; low cost	Poor accuracy; prediction algorithms need optimization, especially for complex movements’ energy expenditure errors	(113, 114, 115)
	Heart Rate Variability (HRV) Models	Time-domain, frequency-domain indicators of heart rate variability	Improves activity recognition and energy expenditure prediction accuracy	Abnormal autonomic function in SCI patients may affect accuracy, requires algorithm or detection adjustments	(117)

Summary of comparison of systematic nutritional assessment methods for patients with spinal cord injury. BIA, Bioelectrical Impedance Analysis; BMI, body mass index; BMR, Basal metabolic rate; CSA, cross sectional area; FFM, Fat-free mass; FFQ, Food Frequency Questionnaire; LBM, lean body mass; LC–MS/MS, liquid chromatography–tandem mass spectrometry; MRI, Magnetic Resonance Imaging; NMR, Nuclear Magnetic Resonance Imaging; REE, resting energy expenditure; SCI, Spinal Cord Injury; TEE, Total energy expenditure; WHR, waist hip ratio; WHtR, Waist-to-height ratio.

### 4.1 Body composition assessment: from macroscopic characterization to precise measurement

Body mass index (BMI) serves as a widely used tool for assessing the body composition of SCI patients, particularly for screening and research purposes (75). While BMI is useful for grouping patients and predicting mortality risk, its limitations in accurately reflecting body composition in SCI patients have been recognized. Studies have linked BMI to mortality risk, with both low (<18.5) and high (≥35.0) BMI associated with increased mortality in SCI patients (76), although higher BMI may offer a slight protective effect over a longer duration (77). BMI’s utility varies depending on the type and extent of spinal cord injury; for example, it is more accurate in paraplegic patients than in those with tetraplegia (78). The substantial reduction in LBM seen post-SCI limits the usefulness of BMI as a sole indicator of obesity and body composition. While adjusting the BMI threshold to 22 kg/m^2^ partially improves obesity screening, it still fails to provide a precise assessment of muscle and fat distribution. To improve accuracy, BMI should be used in combination with other body composition metrics.

Waist circumference, hip circumference, and related measures (e.g., waist-to-hip ratio and waist-to-height ratio) are valuable for assessing body composition, especially for monitoring localized fat (79). Waist circumference, in particular, has been linked to abdominal adiposity, metabolic risk, and the Framingham Risk Score. Studies suggest that a waist circumference cutoff of 94 cm offers 100% sensitivity and 79% specificity in identifying cardiovascular risk in SCI patients, with some refining this cutoff to 86.5 cm (23, 80). These measurements help identify obesity levels and assess the associated risks of cardiovascular disease.

More specific body composition assessments in SCI patients focus on three key areas: muscle, fat, and bone. Macroscopic indicators such as arm and thigh circumference can provide preliminary estimates of muscle mass. When combined with skinfold thickness measurements, they can be used to approximate limb muscle cross-sectional area and body fat percentage in a non-invasive manner (81). However, limb atrophy, especially in quadriplegic and hemiplegic patients, complicates the predictive value of these measurements, necessitating further validation (82). For more precise assessments, imaging techniques such as MRI and ultrasound are increasingly used to measure muscle mass accurately.

The assessment of visceral fat is another important aspect of body composition in SCI patients, as increased visceral fat is closely linked to metabolic diseases such as insulin resistance and type 2 diabetes (83, 84). Visceral fat can be measured using imaging techniques like MRI and CT, which provide precise data on fat distribution, enabling the identification of high-risk individuals and informing personalized interventions.

BMD monitoring is particularly important in the first year following SCI, as this is when bone loss tends to be most severe, especially in weight-bearing regions such as the distal femur and proximal tibia (85). Early monitoring of BMD changes is essential for preventing fractures (85). Body composition monitoring not only helps assess a patient’s physical status and health risks but also guides adjustments in nutritional intake and rehabilitation regimens, supporting the evaluation of interventions such as functional electrical stimulation (86, 87).

Due to the disease-specific nature of SCI, body composition in SCI patients varies over time, depending on the injury type and extent. Monitoring these changes is crucial for developing individualized treatment regimens. Advanced MRI techniques, with their superior soft tissue resolution, allow for accurate quantification of ectopic fat deposition (e.g., hepatic steatosis) and muscle distribution patterns. In SCI populations, such imaging modalities offer distinct advantages by enabling precise characterization of neurogenic obesity and disuse muscle atrophy (24). MRI-derived metrics have already been applied in genome-wide association studies to link body composition traits with metabolic risk (88), and could be similarly valuable in SCI for identifying phenotype-specific metabolic alterations and informing targeted interventions. Ultrasound technology, with its real-time capability, is an effective tool for dynamically assessing muscle mass, particularly in a clinical or rehabilitation setting (89). While dual-energy X-ray absorptiometry (DXA) is considered the ‘gold standard’ for body composition assessment due to its high reproducibility and ability to provide detailed data on fat, muscle, and bone mineral, its use is limited by radiation exposure (79). Bioelectrical impedance analysis (BIA), a non-invasive and easy-to-perform method, is particularly useful for assessing fat mass, offering high accuracy, especially in women and SCI patients with incomplete motor function (90). However, the use of generic BIA prediction equations can lead to inaccurate fat-free mass (FFM) assessments, particularly in male patients with complete cervical cord injury (91). Thus, developing specific BIA equations for the SCI population remains an important area of ongoing research.

### 4.2 Nutritional intake and absorption assessment: from dietary records to metabolomics

Accurate assessment of nutritional intake and absorption is critical for optimizing the health status of patients with SCI. Common methods used to assess nutrient intake include the 24-h dietary recall and the food frequency questionnaire (FFQ). The 24-h dietary recall provides a detailed snapshot of a patient’s food consumption over a short period, offering valuable insights into energy intake and nutrient distribution in SCI patients. It helps reveal dietary patterns and nutrient requirements (92, 93). The FFQ, on the other hand, offers a broader overview of long-term eating habits, helping to assess the overall nutritional status of SCI patients over time (94, 95).

To bridge this gap, the Spinal Nutritional Screening Tool (SNST) was developed. The SNST assesses nutritional status based on eight domains, incorporating SCI-specific indicators such as injury level, pressure ulcer risk, and ventilatory support requirements (96). A total score exceeding 15 suggests high nutritional risk, whereas a score of 10 or below indicates low risk (96). It demonstrates higher sensitivity and negative predictive value in detecting malnutrition risk compared to NST and MUST, particularly among high-risk groups such as tetraplegic patients (96, 97). The tool is suitable for nutritional screening and for use by both clinical dietitians and rehabilitation teams in planning nutritional interventions tailored to SCI-specific challenges.

The Simplified Nutritional Appetite Questionnaire (SNAQ) is a validated instrument that offers a valuable supplement to comprehensive nutritional assessments in individuals with SCI. It consists of several core items targeting appetite, satiety, taste alterations, and eating frequency (98). Due to its brevity and ease of use, the SNAQ is well-suited for both clinical practice and research contexts. Prior studies have shown its utility in effectively evaluating appetite and predicting unintentional weight loss, thereby facilitating the early identification of patients who may benefit from targeted nutritional interventions (99, 100). Combining general and SCI-specific tools such as FFQ, SNST, and SNAQ provides a comprehensive approach to assessing dietary intake and nutritional risk in SCI patients, facilitating timely and individualized interventions.

SCI also causes neurogenic bowel dysfunction and disrupts autonomic regulation, leading to altered nutrient absorption and metabolism. Therefore, it is essential not only to assess nutrient intake but also to monitor nutrient absorption and utilization. Stable isotope labeling techniques, such as ^13^C-leucine, ^15^N-glycine, and ^13^C-glucose, can accurately quantify nutrient absorption efficiency and tissue utilization, offering valuable insights into the metabolic disruptions caused by SCI (101, 102). Furthermore, lactulose/mannitol permeability assays and biomarkers like lipopolysaccharide-binding protein and zonulin can be used to assess intestinal function, providing additional data to understand nutrient absorption and the integrity of the intestinal barrier (103). These methods not only validate dietary self-reports but also incorporate biomarkers to help establish early warning models for nutritional risk in SCI patients (104).

Metabolomics is emerging as a powerful tool for assessing nutrient absorption at the molecular level. By analyzing metabolic profiles, researchers can trace the metabolic fate of nutrients in the body, offering a more detailed understanding of the body’s response to food intake. For example, plasma short-chain fatty acids profiles, analyzed via liquid chromatography–tandem mass spectrometry (LC–MS/MS), can reflect the colonic catabolic efficiency of dietary fiber (105). Additionally, ^1^H nuclear magnetic resonance (NMR) spectroscopy can track changes in serum metabolic profiles over time, helping to observe metabolic shifts during different stages of SCI (106, 107). Studies have identified SCI-specific metabolites, such as acetyl phosphate (linked to delayed carbohydrate uptake) and 1,3,7-trimethyluric acid (reflecting purine metabolism disorders), which not only elucidate the underlying mechanisms of nutrient uptake and metabolism disruptions but, when combined with machine learning, can guide precision nutritional interventions (e.g., macronutrient supplementation, micronutrient fortification, and gut microbiota modulation) for SCI patients (106, 107).

### 4.3 Energy metabolism assessment: static demand and dynamic consumption

SCI patients often experience significant physical dysfunction and metabolic disturbances, leading to energy intake exceeding energy expenditure, which can result in obesity and cardiovascular metabolic disorders. Accurate measurement of energy metabolism is essential to avoid excessive caloric intake and help prevent obesity-related complications. However, predicting energy demands for SCI patients remain challenging due to the large individual variations in this population. Current prediction equations often lead to significant biases (108). Indirect calorimetry (IC), the gold standard for measuring resting energy expenditure (REE), remains the most reliable method for assessing energy needs in SCI patients, but its use is limited by availability and cost (109, 110). Notably, clinical guidelines, including a 2018 consensus on cardiometabolic risk in SCI, recommend the use of IC to estimate energy expenditure and guide nutritional assessment (6).

The doubly labeled water (DLW) technique offers a precise method for evaluating TDEE by tracking the metabolic kinetics of water (111). It is particularly useful in determining changes in physical activity levels but is also complex and costly, which limits its use in large-scale clinical settings (49, 112).

Dynamic energy expenditure monitoring is becoming more feasible through wearable technologies. For patients with incomplete spinal cord injuries, wearable sensors have been developed to estimate energy expenditure, especially for those capable of walking (113, 114). For wheelchair users, accelerometers and demographic data can be used to predict energy expenditure during various activities, such as resting, wheelchair propulsion, and arm movements, offering more accurate predictions of energy needs (29, 114). Smartphone accelerometers have also proven to be a cost-effective alternative for estimating energy expenditure in full-time manual wheelchair users (115). Although commercially available wearable sensors designed for individuals with SCI (e.g., wearable electromyography sensors by Shirley Ryan AbilityLab) are already on the market, their applicability remains limited due to the heterogeneity of the SCI population (116). Challenges persist regarding sensor types, measurement accuracy, adaptability, and cost-effectiveness. Integrating energy expenditure sensors with wearable technologies such as exoskeletons specifically designed for SCI patients may represent a promising direction for future development.

Additionally, heart rate variability (HRV) is gaining attention as a tool for activity recognition and energy expenditure estimation. Studies have shown that HRV parameters can improve the performance of energy expenditure prediction models, suggesting that HRV, combined with wearable technology, has great potential for dynamic energy monitoring in SCI patients (117). However, due to the diversity and complexity of the SCI population, further refinement of algorithms and methods is necessary to enhance the accuracy of energy expenditure predictions for complex activities.

In summary, accurate assessment of energy metabolism is crucial for individuals with SCI, as it directly informs nutritional strategies to prevent obesity and related complications. Despite promising advances in wearable technologies, several challenges persist, including variability in injury characteristics, metabolic heterogeneity, sensor misalignment, and discrepancies between commercial and clinical-grade devices. These factors contribute to inaccuracies in energy expenditure estimation and underscore the need for further algorithm refinement, clinical validation, and device standardization.

Looking ahead, future developments are expected to focus on integrated systems that combine real-time energy monitoring with individualized nutritional feedback. The incorporation of machine learning algorithms and multimodal sensor data—such as accelerometry, heart rate variability, and skin temperature—may enable more precise, personalized metabolic assessments. Cutting-edge innovations include sensor-embedded garments, AI-driven prediction models, and closed-loop systems that automatically adjust dietary plans based on energy outputs (118–120). These technologies not only enhance usability and reduce patient burden but also provide actionable data for clinicians. By supporting dynamic, personalized nutritional management, such wearable systems could play a pivotal role in improving the nutrition-oriented quality of life in SCI patients.

## 5 Conclusion

SCI induces substantial changes in energy balance and nutrient metabolism, which can elevate the risk of obesity, metabolic disorders, and various other complications. Effective management of these alterations requires robust monitoring of body composition, nutrient intake and absorption, and energy metabolism. Integrating advanced assessment tools with personalized interventions is essential for addressing the complex nutritional needs of SCI patients and improving their overall outcomes. Moreover, emerging technologies such as metabolomics and wearable sensors offer promising opportunities to refine clinical practices, enhance the accuracy of nutritional assessments, and support individualized rehabilitation strategies. These innovations have the potential to significantly improve the quality of care for SCI patients and optimize their long-term health and recovery.
